# Pregnancy Tumor in a 31-Year-Old Female with a Facial Port-Wine Stain

**DOI:** 10.1155/2015/472605

**Published:** 2015-12-20

**Authors:** Andrew Rockafellow, Whitney Florin, Elizabeth Philipone, David Koslovsky

**Affiliations:** ^1^Columbia University College of Dental Medicine, New York, NY 10032, USA; ^2^Division of Oral and Maxillofacial Surgery, Columbia University College of Dental Medicine, New York, NY 10032, USA; ^3^Division of Oral and Maxillofacial Pathology, Columbia University College of Dental Medicine, New York, NY 10032, USA; ^4^Private Practice, Metropolitan Oral Surgery Associates, New York, NY 10022, USA; ^5^Division of Oral and Maxillofacial Surgery, Department of Surgery, Weill Cornell Medical Center, New York, NY 10065, USA

## Abstract

Pyogenic granuloma is a type of inflammatory hyperplasia often seen in the oral cavity and occurs in response to stimuli such as local irritants and hormonal factors. Pyogenic granulomas associated with pregnancy are referred to as pregnancy tumors. This report describes the presentation and surgical management of a large pregnancy tumor occurring in a patient with an overlying isolated facial port-wine stain.

## 1. Introduction

Pyogenic granuloma is a nonneoplastic mass of excess granulation tissue that occurs in response to stimuli such as hormonal factors, traumatic injury, or local irritants [1, 2]. Oral pyogenic granulomas are principally found on the gingiva and arise predominantly in females in the second decade of life [2, 3]. Clinical presentation is of an exophytic, lobulated, or smooth surfaced lesion with a red to purplish color and a soft, spongy texture. Surface ulceration is not uncommon [1]. Microscopically, the lesion displays a benign proliferation of endothelial lined vascular channels in an edematous stroma, often with inflammatory cells [1].

Pyogenic granulomas that arise during pregnancy are referred to as pregnancy tumors [1]. A pregnancy tumor has a prevalence of 0.2–9.6% during gravidity [2, 4]. They most commonly appear after first trimester, grow rapidly, and typically regress after delivery [5]. Surgical intervention is often not required. However, pregnancy tumors can be removed during the second trimester if they interfere with occlusion, are painful, bleed excessively, or are excessively large [5]. Lesions excised during pregnancy often recur [5]. After delivery, pregnancy tumors typically recede spontaneously but excision may be necessary for those cases which persist [5].

Yuan et al. described the relationship between pyogenic granulomas and angiogenic factors in pregnancy [6]. According to the authors, female sex hormones not only enhance the expression of angiogenic factors such as basic fibroblast growth factor (FGF) and vascular endothelial growth factor (VEGF) but also decrease cell apoptosis by lessening the expression of tissue necrosis factor-alpha (TNF-alpha) [6]. Women with pyogenic granulomas during pregnancy demonstrated significantly more basic FGF, more VEGF, and less TNF-alpha [6].

Port-wine stain is a congenital capillary malformation with a prevalence of 0.3–0.5% and manifests on the mucosa or skin as pink or red, erythematous patches that can become darker with age [7, 8]. The head and neck region is the most common location, especially in the V1 and V2 dermatomes [7, 8].

The following is a case report of a 31-year-old female with a left facial port-wine stain who developed a large pregnancy tumor of her left maxillary gingiva in a similar distribution to her port-wine stain. The lesion failed to resolve postpartum thus requiring surgical intervention.

## 2. Case Report

A 31-year-old female, two months postpartum, presented to the Oral and Maxillofacial Surgery Department at New York Presbyterian Hospital/Columbia University Medical Center (CUMC/NYPH; New York, NY). The patient's chief complaint was of a large, persistent growth of her left maxillary gingiva that presented during her first trimester of pregnancy. She reported the growth to her obstetrician who was monitoring it and advised her to wait until after her pregnancy before seeking consultation with oral surgery. The patient reported frequent bleeding from the site.

**Figure 1:**
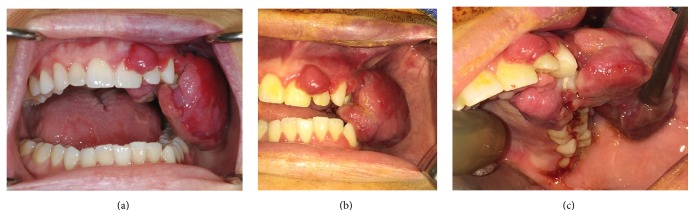
Intraoral frontal photographs at initial presentation with patient in maximum open (a) and closed (b) positions. A red-purple edematous growth is appreciated containing three sublesions: prominent left posterior maxillary buccal, left anterior maxillary buccal, and palatal extensions. The bulk and extent of the lesion prevent the patient from fully occluding. With the lesion retracted (c), the underlying dentition can be appreciated. The port-wine stain of the left upper lip and labial mucosa is evident. The patient is also noted to have geographic tongue as variable sized bald, flat red patches can been seen on the tongue dorsum.

On physical exam, she had a left cheek swelling with a large red-purple lesion of the left maxillary buccal gingiva (Figure 1). The lesion was soft and spongy in texture and easily bled upon manipulation. The lesion extended to the palatal gingiva. The patient also had a left facial port-wine stain, adjacent to the oral lesion, which had been present since birth. The port-wine stain was not limited to the lip, as it involved the facial skin of her left cheek. Intraorally, the left maxillary vestibule displayed hypervascular markings.

Given the clinical appearance of the oral lesion and that it presented during pregnancy, a clinical diagnosis of pregnancy tumor was rendered. Sturge-Weber syndrome, a disorder often associated with facial port-wine stains, had been ruled out due to a lack of other clinical symptoms characteristic of the syndrome, such as neurologic and ocular abnormalities [9–13].

**Figure 2:**
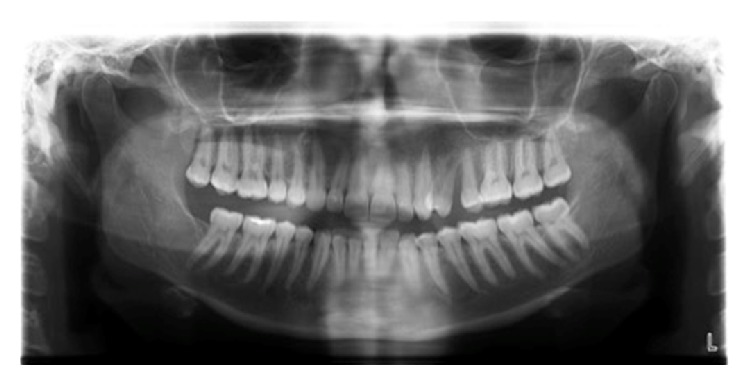
Panoramic radiograph at initial presentation. Interproximal bone loss can be appreciated between teeth numbers 24 and 25 (international nomenclature) and tooth number 24 is noted to be mesially displaced.

A panoramic radiograph depicts interproximal bone loss between teeth numbers 24 and 25 (international nomenclature) and tooth number 24 is mesially displaced (Figure 2). A computed tomography (CT) angiogram revealed a 3.9 × 1.7 cm hypervascular lesion in the left buccal space with a principal arterial blood supply from the left facial branch of the left external carotid artery. No major arteriovenous shunting was demonstrated.

The patient met with an interventional neuroradiologist in preparation for surgery to evaluate the lesion's vascular nature. Preoperative elective embolization of particular branches of the left internal maxillary artery was successful. Branches of the facial artery were spared during embolization to prevent tissue ischemia and necrosis and poor wound healing.

**Figure 3:**
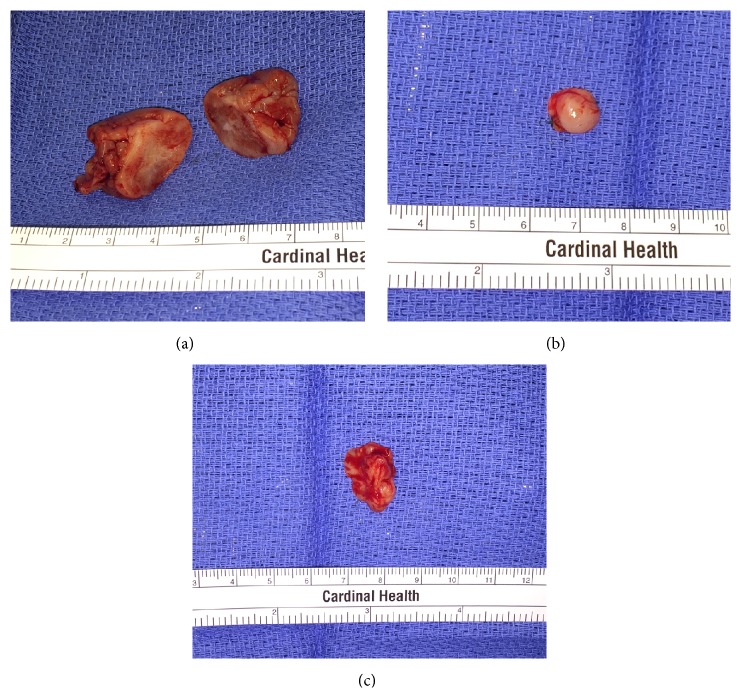
Left posterior buccal (a), anterior buccal (b), and palatal (c) maxillary sublesions. The left posterior buccal sublesion was sectioned for gross examination.

Under general anesthesia, the lesion was excised (Figure 3). The most prominent sublesion of the left posterior maxillary buccal gingiva was excised in full thickness fashion along with a cuff of healthy tissue. The anterior and palatal sublesions were also excised in full thickness fashion along with a cuff of healthy tissue. Although there was significant interproximal bone loss involving teeth numbers 24 and 25 (international nomenclature), they were nonmobile and, therefore, maintained. Peripheral ostectomy was performed to remove any remaining soft tissue in-growths and vascular channels. The flap was closed primarily without tension. The excised specimens were sent for histopathologic review.

**Figure 4:**
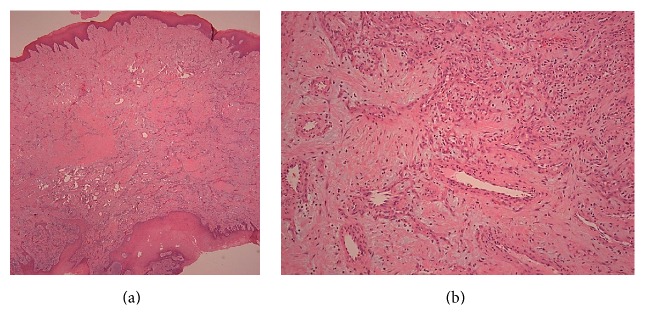
Hematoxylin and eosin (H&E) stain on low power (×20, (a)) magnification reveals numerous vascular channels in a background of fibrous connective tissue. A mixed inflammatory cell infiltrate is scattered throughout the stroma. Surface epithelium is present. On high power (×100, (b)) magnification, thin walled blood vessels are revealed, lined by endothelial cells. The stroma contains fibroblasts and inflammatory cells.

Histopathologic analysis revealed endothelial proliferation, chronic inflammatory cells, and anastomosing vascular channels, all consistent with an oral pyogenic granuloma or pregnancy tumor (Figure 4). Immunohistochemistry staining for GLUT-1 was negative, which ruled out a hemangioma [14].

**Figure 5:**
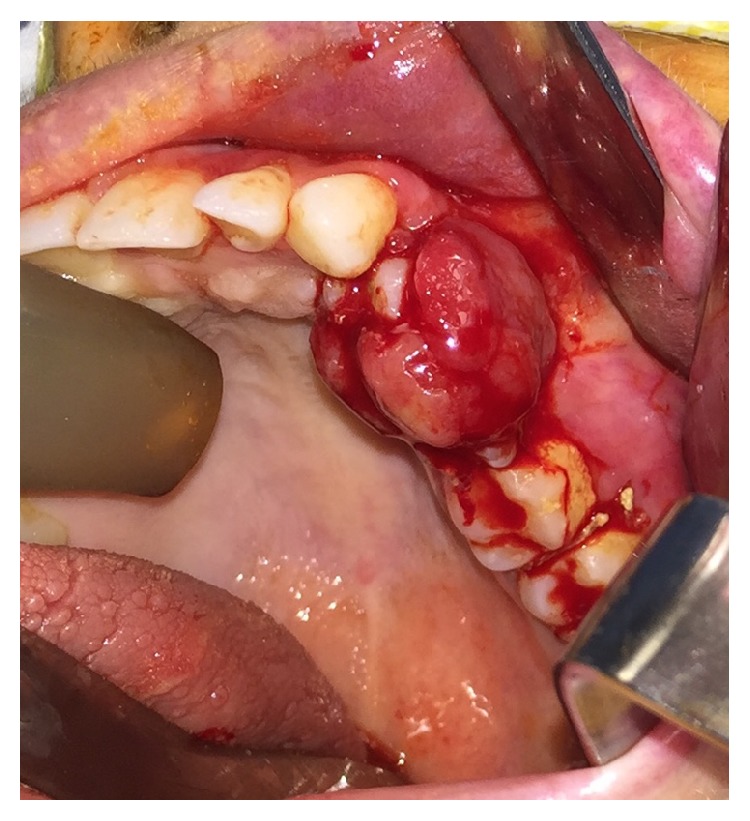
Recurrent lesion two months after initial excision. Teeth numbers 24 and 25 (international nomenclature) are mobile and enveloped by the mass. Tooth number 26 (international nomenclature) is mobile and has a >10 mm probing depth.

**Figure 6:**
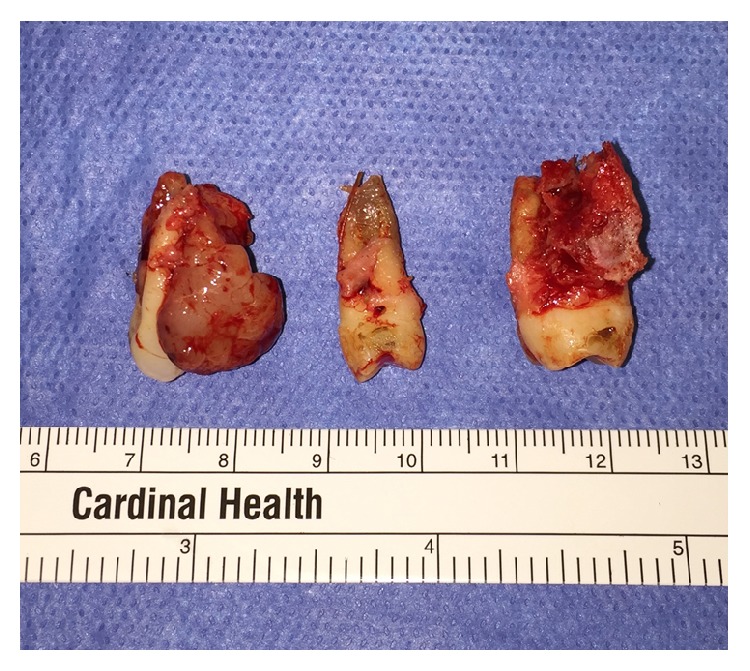
Extracted teeth numbers 24, 25, and 26 (international nomenclature). A portion of the recurrent lesion is seen attached to the distal aspect of tooth number 24 (international nomenclature).

**Figure 7:**
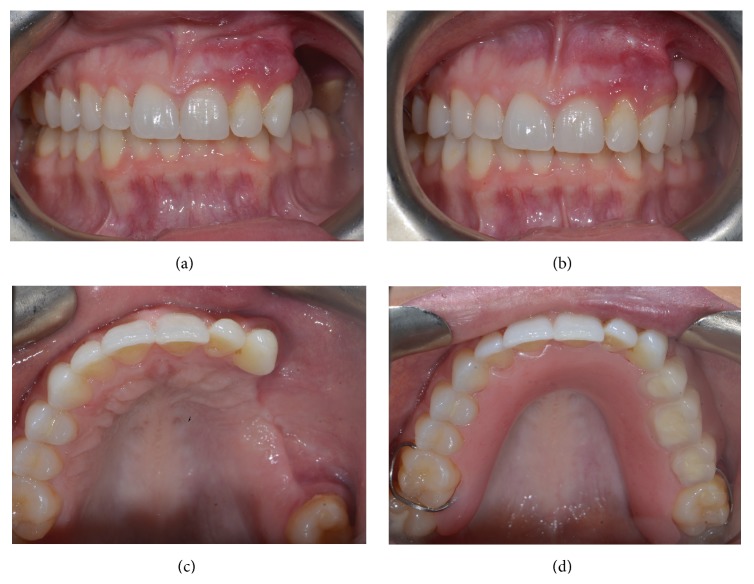
Intraoral frontal ((a), (b)) and occlusal ((c), (d)) views without ((a), (c)) and with ((b), (d)) dental prosthesis. At 11 months after excision of recurrence, there is no evidence of disease. Hypervascular markings persist in the left anterior maxillary vestibule. The removable prosthesis provides esthetic and functional benefits while permitting hygiene and surveillance for recurrence.

The patient was followed closely and initially did well. Three weeks after surgery, the patient complained of new bleeding from the surgical site. Six weeks after surgery, the patient developed a recurrent lesion (Figure 5). Teeth numbers 24 and 25 (international nomenclature) were now grossly mobile and the mesial aspect of tooth number 26 (international nomenclature) displayed a >10 mm probing depth. In preparation for excision of the recurrent lesion, the patient underwent a repeat angiogram. Palatal branches of the left ascending pharyngeal artery, a proximal branch of the left carotid artery, were successfully embolized. Under general anesthesia, the patient underwent excision of the recurrent lesion along with extraction of teeth numbers 24, 25, and 26 (Figure 6). Histopathologic review confirmed a recurrent pyogenic granuloma. The patient was fitted with a removable intermediate prosthesis (Figure 7). The prosthesis provided esthetic and functional benefits while permitting hygiene and surveillance for recurrence.

At most recent follow-up, the patient is 11 months' post-excision of the recurrent lesion and reports being 5 months' pregnant. On monthly examinations, she continues to show no evidence of disease.

## 3. Discussion

The left facial port-wine stain in this patient extended into the mucosa surrounding her pyogenic granuloma. Estrogen and progesterone increase expression of angiogenic factors and decrease granuloma cell apoptosis [6]. Increased blood flow to the region of the pyogenic granuloma through the enlarged capillaries of the port-wine stain may have predisposed the patient to the development of the pregnancy tumor as imaging showed a prominent facial artery [6, 15–17].

The occurrence of cutaneous pyogenic granulomas arising in port-wine stains has been reported in the literature [18–22]. Sheehan and Lesher Jr. conducted a literature search and found 20 cases in addition to their own case of cutaneous pyogenic granulomas arising in port-wine stains [18]. There have also been a number of case reports on cutaneous pyogenic granulomas arising in port-wine stains of patients during pregnancy [19, 23–25]. We were able to locate only one case report of an oral pregnancy tumor occurring within mucosa involved in a port-wine stain [20].

Microscopically, an inflamed hemangioma and pyogenic granuloma prove difficult to distinguish. A tool for differentiating between an inflamed hemangioma and a pyogenic granuloma is a GLUT-1 stain. GLUT-1 is a glucose transport type protein and is undetectable in a pyogenic granuloma but stains positive in an inflamed hemangioma [14].

Management guidelines for granulomas of pregnancy have been reported [26]. If possible, it is often favored to delay intervention until postpartum period since lesions can resolve when hormones stabilize. However, for large lesions that bleed and interfere with function, treatment should be rendered. Various suggested treatment options include curettage, cryotherapy, laser ablation, sclerotherapy, corticosteroid injection, and surgical excision [20–22, 24, 27]. A novel treatment approach utilizing sclerotherapy with sodium tetradecyl sulfate has been described in the literature [20]. The patient consulted with a plastic surgeon to discuss sclerosis via sodium tetradecyl sulfate and refused treatment. The patient also consulted with a cardiologist to discuss the benefits of propranolol but refused treatment. Ultimately, the patient opted for excision as she was two months postpartum and her lesion persisted and was enlarging. Pyogenic granulomas associated with port-wine stains often recur and, therefore, complete surgical excision is generally favored [20, 22].

Preoperative elective embolization was performed to decrease the arterial blood supply and risk for excessive intraoperative bleeding. The embolization was also believed to decrease the abundance of female sex hormones to the region and minimize recurrence. When the lesion recurred, an option for intralesional corticosteroid injection was discussed and rejected by the patient.

Teeth numbers 24, 25, and 26 (international nomenclature) were spared during the first excision and removed during the second surgery. We believe that a potential cause of the recurrence was the inability to remove numerous vascular anastomoses within the interproximal gingiva between the teeth. The ultimate extraction of these teeth allowed for better access and a more complete excision and curettage.

The plan for final skeletal reconstruction and dental rehabilitation may include an implant-supported fixed prosthesis and will be considered if the patient remains disease-free through her current pregnancy.

## 4. Conclusion

This patient's pregnancy tumor, located in the region of a left facial port-wine stain, was determined to be the result of hormonal influences from her pregnancy and vascular anastomoses to the region from the port-wine stain. Pyogenic granulomas in association with port-wine stain can be more resistant to standard treatment. Elective embolization used in tandem with surgical excision is one mode of treatment for these lesions.
